# Low-energy electron interaction and focused electron beam-induced deposition of molybdenum hexacarbonyl (Mo(CO)_6_)

**DOI:** 10.3762/bjnano.13.13

**Published:** 2022-02-04

**Authors:** Po-Yuan Shih, Maicol Cipriani, Christian Felix Hermanns, Jens Oster, Klaus Edinger, Armin Gölzhäuser, Oddur Ingólfsson

**Affiliations:** 1 Carl Zeiss SMT GmbH, Industriestraße 1, 64380 Roßdorf, Germany; 2 Faculty of Physics, Bielefeld University, 33615 Bielefeld, Germany; 3 Science Institute and Department of Chemistry, University of Iceland, Dunhagi 3, 107 Reykjavik, Iceland

**Keywords:** dissociative electron attachment, dissociative ionisation, focused electron beam-induced deposition, molybdenum hexacarbonyl

## Abstract

Motivated by the potential role of molybdenum in semiconductor materials, we present a combined theoretical and experimental gas-phase study on dissociative electron attachment (DEA) and dissociative ionization (DI) of Mo(CO)_6_ in comparison to focused electron beam-induced deposition (FEBID) of this precursor. The DEA and DI experiments are compared to previous work, differences are addressed, and the nature of the underlying resonances leading to the observed DEA processes are discussed in relation to an earlier electron transmission study. Relative contributions of individual ionic species obtained through DEA and DI of Mo(CO)_6_ and the average CO loss per incident are calculated and compared to the composition of the FEBID deposits produced. These are also compared to gas phase, surface science and deposition studies on W(CO)_6_ and we hypothesize that reductive ligand loss through electron attachment may promote metal–metal bond formation in the deposition process, leading to further ligand loss and the high metal content observed in FEBID for both these compounds.

## Introduction

Studies on Mo-based semiconductor materials for the application as thin films with wafer-scale thickness homogeneity [1] and for solar hydrogen production [2] have attracted interest in the last years. For applications of such types a good and target-oriented fabrication control of molybdenum nanostructures is important. Potentially, this may be achievable by focused electron beam-induced deposition (FEBID). In FEBID of metallic structures, organometallic precursor molecules are generally used as the metal source [3–5]. The organometallic precursors are continuously supplied to a substrate surface in proximity to the impact side of a tightly focused, high-energy electron beam in a high-vacuum instrument. Ideally, the organometallic precursor is completely dissociated through the interaction with the high-energy electrons, leaving a pure metal deposit on the surface, while the dissociated, volatile ligands are pumped away [3–7]. However, when high-energy electrons impinge on a substrate surface, these are subject to elastic and inelastic scattering and a significant number of secondary electrons (SEs) are produced [8–9]. Usually, the energy distribution of these SEs has a peak intensity well below 10 eV, with contributions close to 0 eV and falls rapidly off towards higher energies [9–10]. The secondary electrons are very reactive species, and in fact, the decomposition of the precursor molecules in FEBID is primarily attributed to these [8,11]. Furthermore, the spatial distribution of the scattered electrons exceeds that of the focal area of the primary beam causing deposition broadening [8,12] and the incomplete decomposition of the precursors makes composition control difficult [12]. In general, there are four distinct electron-induced processes that lead to molecular fragmentation [13]. These are dissociative electron attachment (DEA), dissociative ionization (DI), neutral dissociation (ND) and dipolar dissociation (DD). The processes differ distinctly in their energy dependence and their efficiency, and they induce different fragmentation patterns. Thus, in order to better understand the performance of individual FEBID precursors, studies on their interaction with low-energy electrons are important.

Metal carbonyls are generally well suited for the use in FEBID as many of these are commercially available, have comparatively high vapor pressure and are fairly stable and easy to handle. Correspondingly metal carbonyl complexes, including the mononuclear Ni(CO)_4_, Cr(CO)_6_, Fe(CO)_5_, W(CO)_6_ and Mo(CO)_6_, have been used as precursors to deposit metals on surfaces through FEBID, partly achieving high metal content in the deposit. A good overview of this work prior to 2008 can be found in Utke and co-workers [3].

In the context of the role of low-energy electron-induced processes in FEBID several studies on DEA and DI of metal carbonyl precursors, including Fe(CO)_5_, W(CO)_6_ and Cr(CO)_6_ have been reported in recent years [14–17]. Earlier studies on the low-energy electron interaction with metal carbonyls include an electron transmission spectroscopy study [18] on W(CO)_6_, Cr(CO)_6_ and Mo(CO)_6_ from the early 1980s, dissociative electron attachment studies on Fe(CO)_5_, W(CO)_6_, Cr(CO)_6_ and Mo(CO)_6_, from Winters et al. (1966) [19], Pignataro et al. (1965) [20] and George et al. (1982) [21] as well as a number of dissociative ionization studies from the same periods [22–24]. For Mo(CO)_6_, the precursor of interest in this study, dissociative electron-capture experiments were reported in the mid-1970s by Winters et al. [19], where the peak positions of negative ions formed through sequential CO loss from one up to four carbonyl groups were reported. Pignataro et al. [20] also published the energy dependence of the same fragment formation from about 0 eV up to about 10 eV and George et al. [21] determined the rate constant for the formation of [Mo(CO)_5_]^−^ and found it to be 1.3 × 10^−7^ cm^3^·molecule^−1^·s^−1^. This is a very high rate constant, which is consistent with cross section measurements for single-ligand loss in DEA to Co(CO)_3_NO [25] and Pt(PF_3_)_4_ [26], which were found to be 4.1 × 10^−16^ cm^2^ and 1.98 × 10^−16^ cm^2^, respectively, that is, only about a factor of 10 below the πλ^2^, s-wave attachment cross section given by the respective DeBroglie wavelength [26–27]. Using the approximation 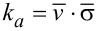 [28], where, *k*_a_ is the rate constant, 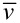, the average velocity of the incident electron and 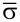 is the respective average cross section. These translate to rate constants of ca. 2.43 × 10^−8^ and ca. 1.2 × 10^−8^ cm^3^·molecule^−1^·s^–1^, respectively, at 1.0 eV electron energy (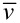 = 5.93 × 10^5^ m/s), approximately where the maxima for these processes are observed.

Electron impact ionization of Mo(CO)_6_ has been reported by a number of groups in the past [22–24,29–31] also including the appearance energies for the loss of one to six CO ligands upon electron impact [22–24,29–30]. Further determination of the ionization energy of Mo(CO)_6_ has been reported from photo-electron [32–35] and two photo-ion studies [36–37].

In the present work, we revisit low-energy electron interactions with Mo(CO)_6_. We present DEA ion yield curves in the energy range from about 0 to 12 eV and compare these to the earlier studies and discuss the DEA contributions in relation to the underlying resonances. Thermochemical threshold energies are calculated at the DFT level of theory for carbonyl loss from Mo(CO)_6_ through DEA and discussed in relation to the current and previous studies. We present EDX analyses of the composition of FEBID deposits from Mo(CO)_6_ and compare the deposits composition with carbonyl loss through DEA vs DI. We compare this to previous gas phase, surface science and FEBID experiments on W(CO)_6_ and discuss these studies in context to the current findings and potential deposition mechanisms.

## Method

### Quantum chemical calculations

Similar to the approach in [38], thermochemical thresholds for the formation of negative ions through DEA were calculated using ORCA [39]. Geometry optimizations and single-point energy calculations were performed at the PBE0 (hybrid GGA functional) [40–41] PBE0/ma-def2-TZV [42–43] level of theory. The Def2 effective core potential [44] for the molybdenum core electrons was used, including the D3(BJ) dispersion correction by Grimme and co-workers [45–46]. The PBE0 functional was chosen as it has been reported to be among the best performers in thermochemical studies on transition metal compounds [47–49]. Harmonic vibrational frequencies were calculated at the same level of theory. They were confirmed to be positive and were used to derive zero-point vibrational energy and thermal energy corrections. Potential alternate spin states were investigated in order to make sure that the lowest energy state was indeed determined for each fragment.

### Experimental

#### Gas phase

Both negative and positive ion yield curves and mass spectra were recorded with an electron–molecule crossed beam setup. The apparatus has been previously described in detail [50] and thus only a brief description is given here. It consists of a trochoidal electron monochromator (TEM), an effusive gas inlet system and a quadrupole mass spectrometer (Hiden EPIC1000). A quasi mono-energetic electron beam generated by the TEM, crosses an effusive beam of the target molecules in a collision region confined by the extraction optics of the quadrupole mass spectrometer (QMS). Both positive and negative ions resulting from the electron–molecule interaction are analyzed and detected using the quadrupole mass spectrometer. The flow of the molecular beam can be controlled with a leak valve. The TEM is heated to 120 °C with two halogen lamps in order to avoid condensation of the target gas on the electrical lens components. The base pressure in the chamber was around 5 × 10^−8^ mbar. The effusive beam of the target gas was generated by sublimation of solid Mo(CO)_6_ at room temperature and the target gas pressure inside the chamber was kept in the range of 8 × 10^−7^ to 1 × 10^−6^ mbar during measurements. Positive ion mass spectra were recorded by scanning through the relevant *m*/*z* range at fixed electron energy while negative ion yield curves were recorded at fixed *m*/*z* by scanning through the relevant electron energy. The electron energy scale was calibrated based on the SF_6_^−^ formation from SF_6_ at 0 eV and the energy resolution was estimated from the full width at half maximum (FWHM) of that signal and was found to be ca. 150 meV.

#### Deposition and EDX analysis

Focused electron beam-induced deposition was performed with a ZEISS photomask repair tool MeRiT^®^. Energy-dispersive X-ray analyses was done with a ZEISS Crossbeam system with an integrated Oxford EDX detector. Deposits for EDX analysis were prepared on a 100 nm thick polycrystalline gold substrate on a 4 inch silicon wafer, purchased from Georg Albert PVD. The Au-based substrate was chosen because of its distinguishable EDX peak among the Mo, C and O elements at 5 keV primary beam energy. Cleanliness of the surface was probed by EDX prior to measurements, confirming the absence of C and O at the surface within the s/n level of the measurements. An electron dose of 8.33 × 10^13^ was applied on a scanning area of 1 μm^2^, resulting in a 230 nm thick pad. Energy-dispersive X-ray analysis was carried out at 5 keV beam energy.

Mo(CO)_6_ was purchased from Merck, Darmstadt, Germany, with stated purity of 98% and used as delivered.

## Results and Discussion

### Dissociative electron attachment

**Figure 1:**
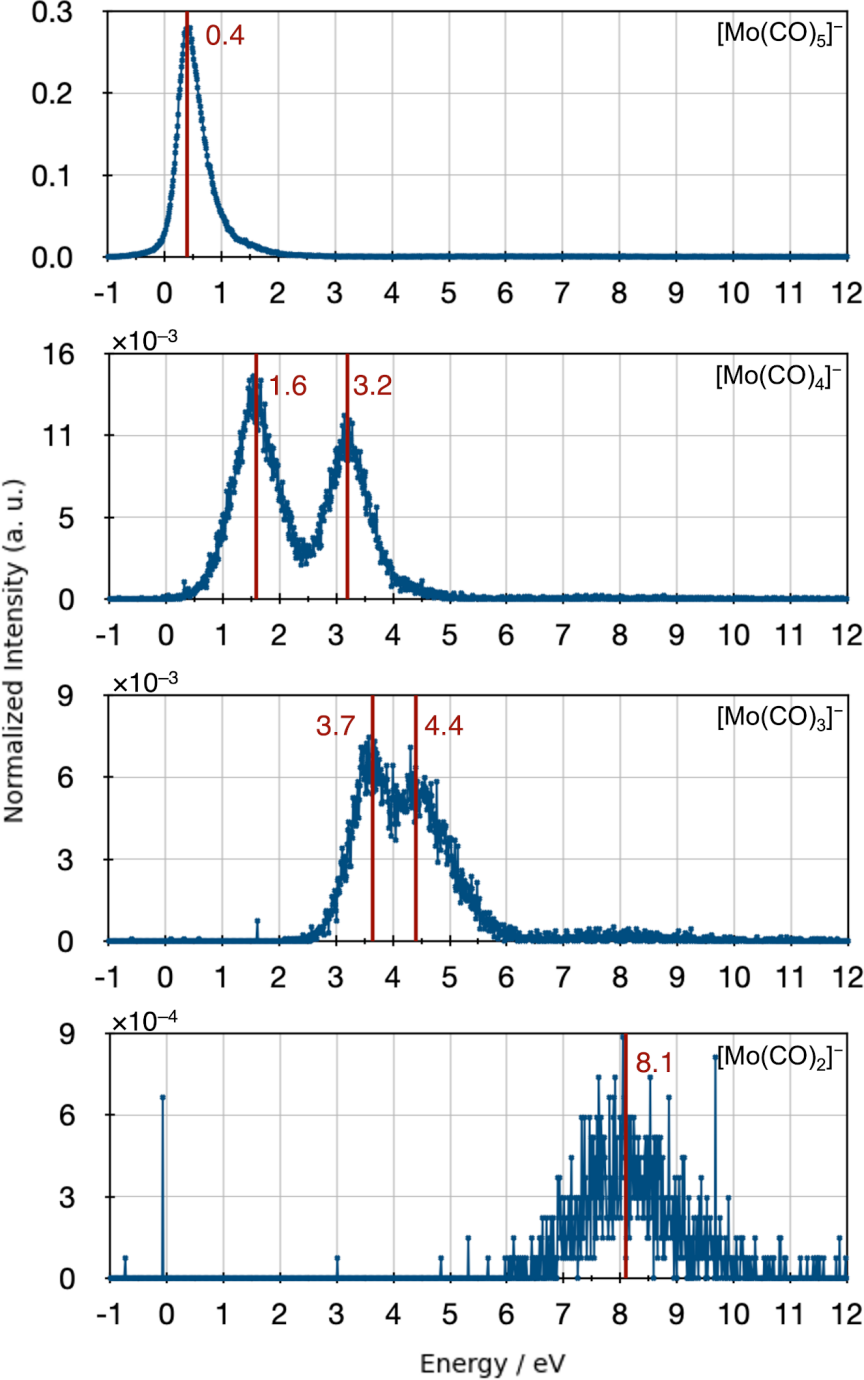
Negative ion yield curves from DEA to Mo(CO)_6_ in the energy range from about 0–12 eV. From top; formation of [Mo(CO)_5_]^−^ (*m*/*z* 236), [Mo(CO)_4_]^−^ (*m*/*z* 208), [Mo(CO)_3_]^−^ (*m*/*z* 180) and [Mo(CO)_2_]^−^ (*m*/*z* 152).

Figure 1 shows the ion yield curves of the negative ions formed upon electron attachment to Mo(CO)_6_. Under the current experimental conditions we observed the formation of four anionic fragments, [Mo(CO)_5_]^−^, [Mo(CO)_4_]^−^, [Mo(CO)_3_]^−^ and [Mo(CO)_2_]^−^. The molecular ion [Mo(CO)_6_]^−^ was not observed. This is consistent with previous studies [19–20]. The four dissociation channels describe the series of loss of the CO ligand. As also observed for W(CO)_6_ [15,19–20], but in contrast to Cr(CO)_6_ [16–17,19–20] and Fe(CO)_5_ [51], the series of CO loss is not complete [19–20] and there is no formation of the bare metal anion. The intensities of the DEA curves in Figure 1 reflect the relative efficiencies of these reaction channels normalized to the intensity of the formation of SF_6_^−^ from SF_6_ and the respective target gas pressure during each specific experiment. The values are given in arbitrary units and we note that ion extractions efficiency, transmission through the QMS or the energy dependence of the electron current are not considered. The most favorable DEA process is the formation of [Mo(CO)_5_]^−^, that is, removal of one carbonyl group, at close to thermal electron energy. The intensity of the DEA ion yield curves decreases with the number of carbonyl groups dissociated, which is in agreement with what has been reported in previous studies on DEA of metal carbonyls [15–17,19–20]. This is shown in Table 1 along with the peak maxima observed in the ion yields for individual fragments in the current study and those reported by Winters and Kiser [19] and by Pignataro and co-workers [20]. The peak values reported from Pignataro and co-workers were extracted from their ion yield curves as they only report the values for the appearance energies, but the ion yield curves are shown for all fragments. Table 1 also shows the calculated threshold values for the individual processes at the PBE0/ma-def2-TZVP level of theory. Qualitatively the ion yield curves observed in the current study offer the same overall picture as reported in the earlier studies, but there are also differences. The peak attributed to [Mo(CO)_5_]^−^ is in the current study and in the study by Pignataro and co-workers [20], observed at an energy of 0.4 eV and not at 2.2 eV as by Winters and Kiser [19]. Resonant electron capture at threshold is clear in the electron transmission spectra from Giordan et al. [18] indicating that in the study by Winters and Kiser [19] this might be an artifact rooted in lack of low-energy electrons from the ionization source of the mass spectrometer used in their study. This is further supported by the fact that the peak maxima for single CO loss from other metal carbonyls reported by Winters and Kiser are all close to 2 eV, while more recent studies [15–17] and the study by Pignataro and co-workers [20] report single CO loss from these compounds close to 0 eV. A further indication is in the relative abundance ratios of 100:65 reported by Winters and Kiser [19] for [Mo(CO)_5_]^−^ vs [Mo(CO)_4_]^−^, while these are closer to 20:1 in the current study. This implies that [Mo(CO)_5_]^−^ observed at 2.2 eV by Winters and Kiser, is from the high-energy tail of the threshold resonances or from the 1.65 eV resonance observed in the transmission spectra reported by Giordan and co-workers [18]. For the loss of two CO ligands all studies report a clear contribution peaking at around 3 eV; however, in the current study an additional contribution is observed at about 1.6 eV. For the loss of three CO groups two contributions are observed in the current study (3.7 and 4.4 eV) and in the study by Pignataro and co-workers [20] (3.9 and 4.7 eV), while only one contribution at 4.1 eV is reported by Winters and Kiser [19]. Finally, all three studies observe the loss of four CO groups through a broad contribution at fairly high energy (8.1, 7.5 and 10 eV, respectively). In the electron transmission spectroscopy study by Giordan et al. [18], electron capture is observed close to 0 eV and at least two more contributions are observed below 1 eV. These are tentatively assigned to the 11t_1u_, 5t_2g_ and 7e_g_ orbitals, though it is pointed out that the structures observed may in part reflect the 0.25 eV vibrational frequency of CO in hexacarbonyls [18] from one electronic state. The contributions at 1.65, 2.14 and 3.29 eV are assigned to the higher lying 3t_2u_, 8e_g_ and 6t_2g_ orbitals, respectively. These assignments are based on Xα calculations and the authors explicitly point out that the assignments of the peaks observed are “far more complex than any spectra of this type previously presented” [18].

**Table 1:** Experimentally determined peak positions for fragments observed in dissociative electron attachment to Mo(CO)_6_, compared to literature values [19–20] and the respective thermochemical threshold values calculated at PBE0/ma-def2-TZVP level of theory.

Anion	Calculated threshold PBE0/ma-def2-TZVP (eV)	Ion yield peaks current study (eV)	Ion yield peaks [19] (eV)	Ion yield peaks [20] (eV)

[Mo(CO)_5_]^−^	−0.2	0.4	2.2	0.4
[Mo(CO)_4_]^−^	1.8	1.6 and 3.2	3.0	3.0
[Mo(CO)_3_]^−^	3.9	3.7 and 4.4	4.1	3.9 and 4.7
[Mo(CO)_2_]^−^	6.6	8.1	7.5	10

**Figure 2:**
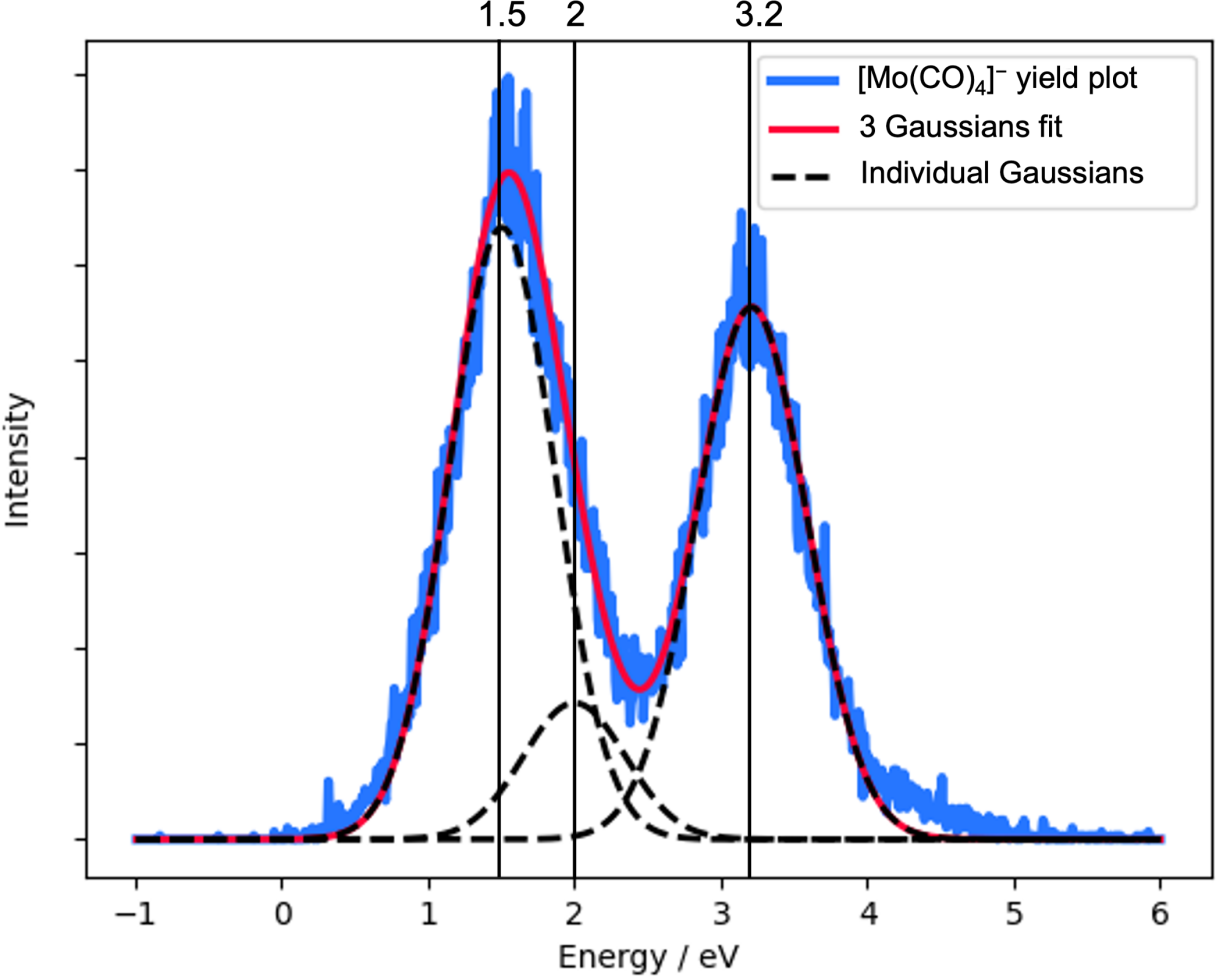
Fit to the [Mo(CO)_4_]^−^ ion yield using three Gaussian functions to represent the 1.65, 2.14 and 3.29 eV resonances observed in the ETS from Giordan et al. [18] (*R*^2^ = 0.98).

The contributions appearing in the current study at 1.6 eV and at 3.2 eV in the ion yield of [Mo(CO)_4_]^−^ correspond well with the resonances observed at 1.65 eV and at 3.29 eV in the electron transmission study. The contribution at 3.7 eV in the [Mo(CO)_3_]^−^ ion yield can be attributed to the high-energy tail of the latter of these two resonances. In general, fragment formation in DEA is shifted to lower energies with respect to the underlying resonances. This is due to the inherent competition between auto-detachment and dissociation of the respective temporary negative ions (TNIs), whereby the survival probability with respect to dissociation is largest at low energies [13]. This assignment of the 1.6 and 3.2 eV contributions in the [Mo(CO)_4_]^−^ ion yield and the 3.7 eV contribution in the [Mo(CO)_4_]^−^ ion yield to the 1.65 and 3.29 eV resonances observed in the electron transmission experiments leaves the contribution of the 2.14 eV resonance unaccounted for in the ion yields. This may be due to the short lifetime of this resonance and/or the fact that [Mo(CO)_4_]^−^ formed through this resonance is masked by the higher-intensity contributions at 1.6 and 3.2 eV. This is demonstrated in Figure 2, which shows a reproduction of the [Mo(CO)_4_]^−^ ion yield by the fit of three Gaussian functions with peak positions slightly below that of the respective resonances. Finally, the [Mo(CO)_3_]^−^ ion yield at about 4.4 eV cannot be attributed to a distinct feature in the ETS and the low-intensity, high-energy [Mo(CO)_2_]^−^ contribution is outside the energy range covered by the ETS.

An alternative assignment of the observed DEA contributions to the resonances observed in the ETS would be that the 2.14 eV resonance appears through the 3.2 eV contribution in the [Mo(CO)_4_]^−^ ion yield and the 3.29 resonance through the 4.4 eV contribution in the [Mo(CO)_3_]^−^ ion yield. This would, however, require the DEA ion yields to be shifted to higher energies with respect to the underlying resonances. Such a situation may occur when the thermochemical threshold for the respective channel is above the resonance maxima or when the immediate precursor is metastable and the observable fraction of its fragmentation products falls preferably within the high-energy tail of the underlying resonance. However, the observation of the 1.6 eV contribution in the [Mo(CO)_4_]^−^ and the calculated threshold energy of 1.8 eV for the formation of this fragment show clearly that the thermochemical threshold for its formation is well below the 2.14 eV resonance.

In addition to the DEA peak values, Table 1 shows the thermochemical thresholds for the formation of [Mo(CO)*_n_*]^−^ (*n* = 1–5), calculated at the PBE0/ma-def2-TZVP level of theory and corrected for the thermal energy at room temperature (25 °C). From these values it is clear that the formation of [Mo(CO)_5_]^−^ is exothermic. Though the calculated thresholds for the loss of two and three CO groups are slightly higher than the experimental peak values for the lower-energy contributions in the respective ion yields, the calculations offer qualitatively a consistent picture of the thermochemistry observed, that is, the shift to higher energies with increasing CO loss. Furthermore, the ion extraction time in our instrument is around 10 μs and the flight time through the quadrupole mass filter is about 100 μs. Thus, only ions are observed that fragment within the first 10 μs and are stable enough to survive the 100 μs flight through the mass filter. This explains the absence of any high-energy contributions in the [Mo(CO)_5_]^−^ yield as, within the current observation time, ions with internal energy that exceeded the threshold for the fragmentation of a second CO group fragment further to form [Mo(CO)_4_]^−^. Similarly, the contribution at 4.4 eV in the [Mo(CO)_3_]^−^ yield is as good as absent in the [Mo(CO)_4_]^−^ yield.

### Dissociative ionization

**Figure 3:**
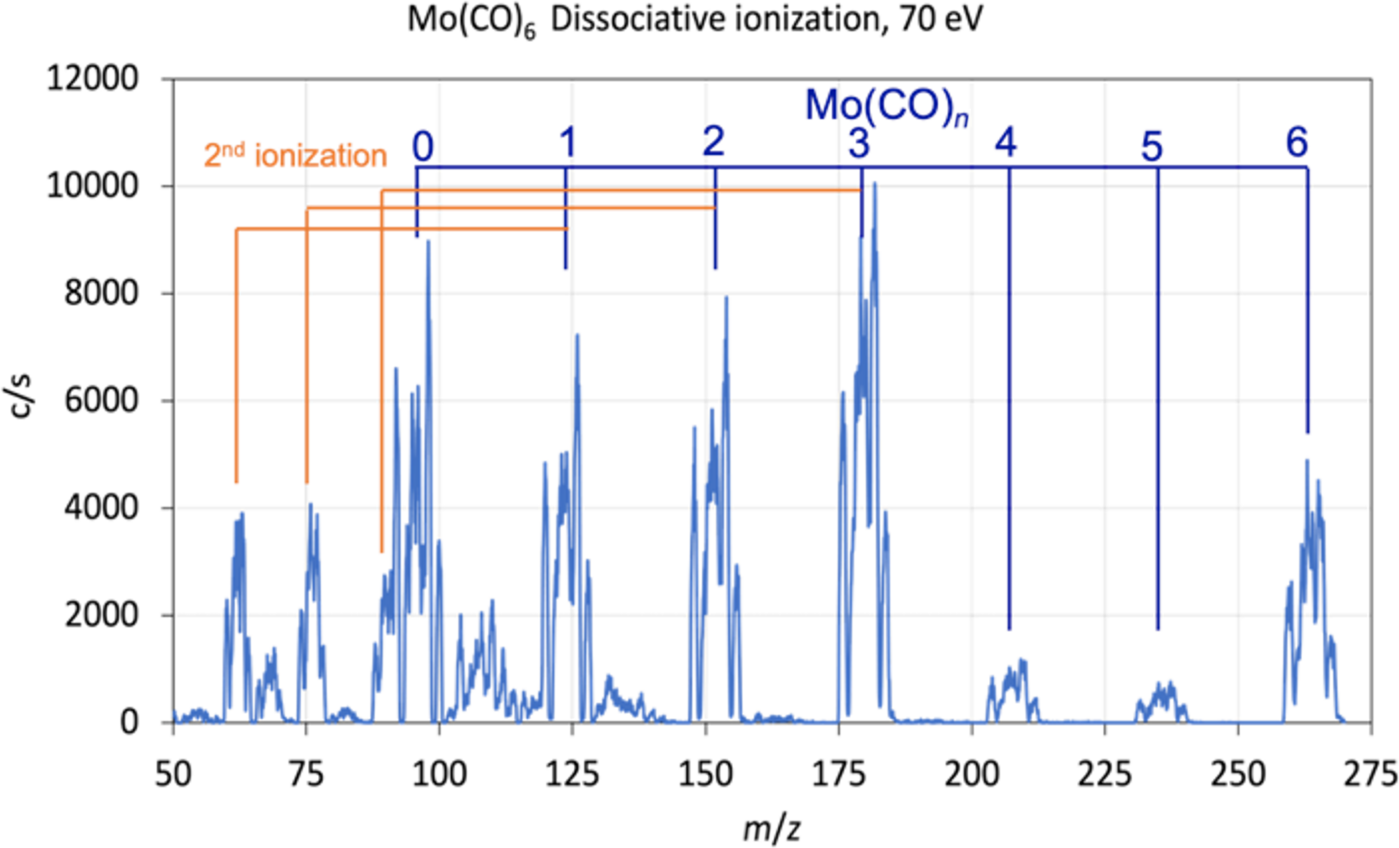
Electron impact ionization mass spectrum for Mo(CO)_6_ recorded at 70 eV incident electron energy with the sequential carbonyl loss (one to six CO groups) and second ionizations marked with vertical lines.

Figure 3 shows an electron impact ionization mass spectrum of [Mo(CO)_6_] recorded at an incident electron energy of 70 eV. This incident electron energy was chosen for ease of comparison as electron impact ionization mass spectra are conventionally reported at this energy. Generally, this is close to the maximum cross section for all DI channels, but at lower energies, close to the threshold region, the relative contributions will be determined by the energy dependence of the onset of individual channels. The mass spectrum is characterized by the broad isotope distribution of molybdenum. It shows the formation of the molecular ion and a progression of CO ligand loss upon electron impact. In this progression, the loss of all six CO groups, that is, the formation of the bare metal ion, Mo^+^, is also observed with appreciable intensity. Such sequential CO loss is commonly observed in dissociative ionization of metal carbonyls; however, the loss of all ligands is generally less efficient for other organometallic species [52–53]. The doubly ionized species [Mo(CO)*_n_*]^++^, with *n* = 1, 2 and 3, are also observed with fairly significant intensity. Contributions which we attribute to [MoC(CO)*_n_*]^+^ with *n* = 0, 1, 2 and 3, that is, molybdenum carbide and the molybdenum carbonyl carbides with one to three CO ligands still attached, are also observed. However, the intensities of the carbide signals for *n* = 2 and *n* = 3 are very low. Finally, contributions are also observed at around *m*/*z* 54, 69 and 82, which correspond to the doubly charged molybdenum carbide and the doubly charged molybdenum carbide with one and two CO groups attached, respectively. All these contributions agree well with the spectra reported at the NIST Chemistry WebBook [54].

Noticeably, the relative intensity of [Mo(CO)_5_]^+^ and [Mo(CO)_4_]^+^ is comparatively low, reflecting further CO loss from these ions within the time frame of our observations. In an ionizing electron collision substantial excess energy is left in the ionized species. This leads to fragmentation, which in the case of metal carbonyls is mainly characterized by sequential CO loss. As mentioned above, in the current experiment the ion extraction time is of the order of 10 μs and the flight time through the quadrupole mass filter is about 100 μs. The ions observed in the mass spectrum are those that are formed during the extraction time and are stable during their flight through the mass filter. This explains the comparatively low intensity of [Mo(CO)_5_]^+^ and [Mo(CO)_4_]^+^, which apparently undergo further CO loss outside the observation window of our instrument.

### Dissociative ionization, dissociative electron attachment and FEBID composition

Table 2 compares the relative contributions of individual ionic species observed in DEA and DI of Mo(CO)_6_. Similar to the approach in [55–57], the relative DEA contributions were obtained by integrating the negative ion yield over the whole width of individual contributions in the ion yield curves for each anionic fragment. These were then normalized to the [Mo(CO)_5_]^−^ contribution, which is set to 1. The relative DI contributions of the observed cationic fragments were obtained by integrating over the isotope distribution for each Mo-containing fragment in the positive ion mass spectrum and normalizing that to the integral value of the parent molecular ion peaks, which is set to 1. Table 2 also reports the average CO loss for DEA and DI per incident, calculated by taking the sum of the products of the relative contribution for each Mo-containing fragment times the number of CO groups lost and dividing that by the sum of the relative contributions of all Mo-containing fragments. Using this approach, we found that DEA leads to an average weighted CO loss per incident of about 1.2 while DI leads to an average weighted CO loss of 3.7.

**Table 2:** Relative yields of Mo-containing ions formed by DEA and DI to Mo(CO)_6_ and average weighted CO loss per DEA and DI incident.

Fragment	*m*/*z*	Relative DEA yield	Relative DI yield

[Mo(CO)_6_]^+^	264	—	1
[Mo(CO)_5_]^+/−^	236	1	0.15
[Mo(CO)_4_]^+/−^	208	0.13	0.24
[Mo(CO)_3_]^+/−^	180	0.066	2.01
[Mo(CO)_2_]^+/−^	152	0.0047	1.4
[MoCO]^+^	124	—	1.25
MoC^+^	108	—	0.43
Mo^+^	96	—	1.27
[Mo(CO)_3_]^++^	90	—	0.4
[Mo(CO)_2_]^++^	76	—	0.48
[MoCO]^++^	62	—	0.46

Average weighted CO loss		1.23	3.7

**Figure 4:**
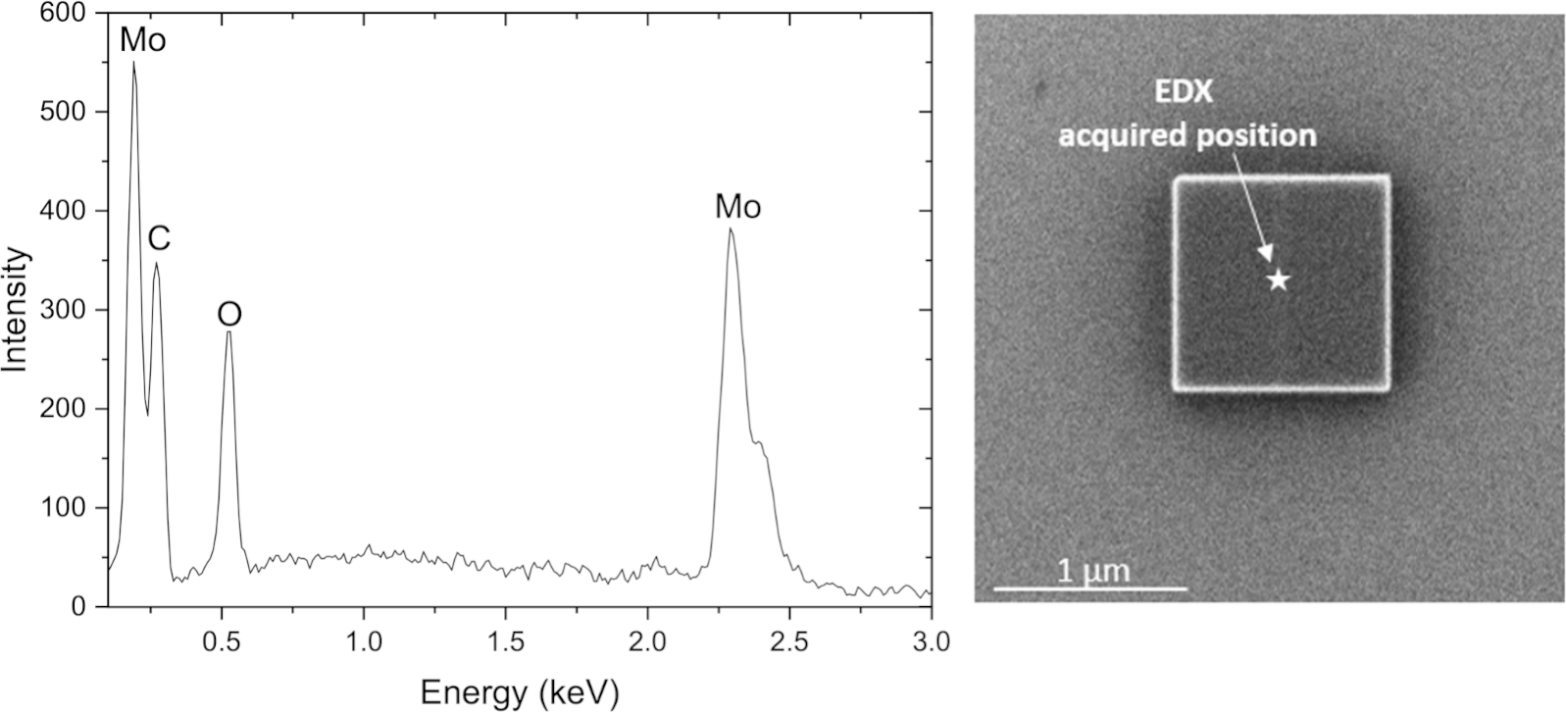
Left: EDX spectrum. Right: SEM image of FEBID pad (1 μm^2^) Mo(CO)_6_.

For comparison FEBID deposits were created on a polycrystalline gold substrate allowing for a clear distinction between EDX peaks from the Mo, C and O elements from the original precursor. Deposition was carried out with an electron dose of 8.33 × 10^13^ applied on a scanning area of 1 μm^2^, which resulted in a 230 nm thick pad. EDX measurements were carried out with a ZEISS SEM system with an integrated Oxford EDX detector. The elemental composition of Mo(CO)_6_ FEBID deposits obtained by EDX measurement is shown in Table 3. Figure 4 shows the corresponding EDX spectrum along with the SEM image of the deposit.

**Table 3:** Elemental composition of Mo(CO)_6_ FEBID deposits obtained by EDX measurement of a 1 μm^2^ pad deposited on a 100 nm thick polycrystalline gold substrate on a 4 inch silicon wafer.

Element	Atom %

C	37.7
Si	0.6
O	20.5
Mo	41.1
Au	0.1
Total:	100

Traces of the EDX signal are discernable from the Au and Si components of the substrate, but the Mo, C and O signals dominate the contributions with 41.1, 37.7 and 20.5 atom %, respectively. This translates to an approximate elemental Mo/C/O composition of 2:2:1 as compared to the initial elemental composition of Mo(CO)_6_ of 1:6:6. We attribute the higher carbon content of the deposit as compared to oxygen to electron-induced ligand decomposition similar to what has been observed in a surface study on W(CO)_6_ [58] and is discussed here below. Carbide formation in the initial dissociation process may contribute, but judging from the gas phase DI experiments this contribution alone should not make the carbon content more than 5% higher than that of oxygen. Carbon deposition from the background gas was also considered, but EDX measurements of the surface exposed to the same electron dose as in the Mo(CO)_6_ deposition experiments showed insignificant carbon deposition, ruling out the background gas as the source of additional carbon in the deposit as compared to oxygen. The 2:1 Mo/O composition puts the average CO loss at 5.5 CO per Mo(CO)_6_ deposited, while the 1:1 Mo/C composition puts the average CO loss at 5.0 CO per Mo(CO)_6_. This is significantly higher than the CO loss per DEA incident observed in the gas phase experiments and also higher than the CO loss per DI incident observed in these experiments. This is very different from the observations made by Rosenberg et al. [58] when comparing the CO loss from few monolayers of W(CO)_6_, adsorbed onto cooled gold substrates and exposed to 500 eV electrons, with the respective gas phase experiments. In that study a weighted average CO loss through DEA of ca. 2 was estimated and a DI average weighted CO loss of ca. 4 [12]. In the surface study on W(CO)_6_, significant initial CO desorption was observed, which decreased rapidly with increased electron dose and was already insignificant above 1 × 10^17^ e^−^/cm^2^. An XPS analysis of the remaining deposit revealed an average loss of two CO groups from the W(CO)_6_ adsorbate at about 7 × 10^16^ e^−^/cm^2^, but above that dose the dominating pathway becomes electron-induced CO decomposition leading to the formation of W(VI) oxide and graphite. This is consistent with an initial DEA step rather than DI leading to partial CO loss while the further, slower decomposition lacks the loss and desorption of intact ligands and is rather dominated by ligand decomposition through DI and surface-induced processes. The secondary electron energy distribution is not largely influenced by the primary electron energy [10]. However, in a W(CO)_6_ FEBID deposition at 5 keV, Mulders et al. [59] reported close to 37 atom % of W and C and about 27% O at room temperature. At about 150 °C, they reported about 55 atom % W, 25 atom % C and close to 15 atom % O. At room temperature the reported values translate to similar CO loss as observed in the current study on Mo(CO)_6_, that is, more than five CO groups are lost on average from each precursor molecule in the process of the deposit formation. Reductive elimination of d^8^ and d^6^ metal complexes is well-known [60–62] and the formation of multiple metal–metal bonds replacing the ligand field stabilization lost in this process has been shown for a number of dinuclear Mo and W coordination compounds [62]. In principle, electron attachment represents reduction of the respective compound while ionization through electron removal represents oxidation. Thus, if sufficient proximity is provided, DEA as an initial fragmentation step should provide the prerequisite for metal–metal bond formation and nucleation points for further CO loss as is shown schematically in the Graphical Abstract accompanying this article. Though admittedly speculative, this may explain the very different findings in surface science studies. The surface science studies are conducted under non-steady-state conditions, where few monolayers are exposed to 500 eV electrons from a flood gun, while the FEBID experiments are conducted with a focused electron beam at 5 keV under steady-state conditions, where continuous replenishment of precursors on a freshly exposed surface is provided. Furthermore, while the surface studies of W(CO)_6_ deposited on a gold surface were conducted at about 160 K, the FEBID studies on this precursor were conducted at elevated temperatures and at room temperature while the current FEBID with Mo(CO)_6_ was conducted at room temperature. At these higher temperatures the mobility at the surface is higher and conceivable activation barriers associated with the metal–metal bond formation will be more easily surmounted.

## Conclusion

Here we have reported on the interactions of low-energy electrons with the FEBID precursor Mo(CO)_6_ and on the composition of deposits made with this precursor. Dissociative electron attachment in the energy range from about 0 up to 12 eV leads to the loss of up to four CO ligands, but neither the molecular parent anion nor the bare metal anion are observed within the detection limit of our experimental set up. The dominant DEA channel is the formation of the anionic fragment [Mo(CO)_5_]^−^ through a low-energy contribution close to 0 eV electron energy and further CO loss is observed through distinct contributions at higher energies. Dissociative ionization, in contrast, leads to a more effective ligand loss and complete CO loss, doubly charged fragments and the formation of carbide are also observed at 70 eV impact energy. Our DEA results are in qualitative agreement with previous studies [19–20] and offer clarification of previous disparities. With respect of the efficiency of DEA as compared to DI, we find that the average CO loss per incident in DEA is about 1.2 while the average loss trough DI is about 3.7. FEBID of Mo(CO)_6_ leads to deposits with an approximate Mo/C/O composition of 2:2:1 as determined by EDX. Using the Mo/O ratio (2:1) as a marker for the CO loss this corresponds to an average loss of about 5.5 CO groups per precursor molecule, which is significantly higher than would be expected from either DEA or DI. We have discussed these findings in connection with electron-induced decomposition of W(CO)_6_ under non-steady-state conditions and its deposition in FEBID with a 5 keV beam. Similar to Mo(CO)_6_, significant CO loss is observed in the FEBID experiments for W(CO)_6_ [59]. Non-steady-state electron exposure, in contrast, shows rapid loss of only two CO groups on average, which in conjunction with gas phase experiments was attributed to an initial DEA step governing the electron-induced decomposition [12]. Here we hypothesize that this may also be the first step in the FEBID process, promoting reductive ligand elimination and metal–metal bond formation. We anticipate that non-steady-state electron exposure of Mo(CO)_6_ would reveal similar mechanisms as observed for W(CO)_6_.
